# Input data resolution affects the conservation prioritization outcome of spatially sparse biodiversity features

**DOI:** 10.1007/s13280-023-01885-6

**Published:** 2023-06-02

**Authors:** Topi Tanhuanpää, Ninni Mikkonen, Heini Kujala, Einari Heinaro, Janne Mäyrä, Timo Kumpula

**Affiliations:** 1 Department of Geographical and Historical Studies, University of Eastern Finland, Yliopistonkatu 7, 80101 Joensuu, Finland; 2 Finnish Environment Institute SYKE, Latokartanonkaari 11, 00790 Helsinki, Finland; 3 Finnish Natural History Museum, University of Helsinki, Pohjoinen Rautatiekatu 13, P.O. Box 17, 00014 Helsinki, Finland; 4 Department of Forest Sciences, University of Helsinki, Latokartankonkaari 7, P.O. Box 27, 00014 Helsinki, Finland

**Keywords:** Conservation prioritization, Forests, Remote sensing, Scale, Spatial resolution, Zonation

## Abstract

Detailed spatial data are an essential part of land use planning and decision-making. Their spatial resolution sets limitations to their use, as coarse datasets are not suitable for detecting small-scale phenomena. In this study, we explored the effects of spatial resolution on the ecological outcome of a conservation prioritization process in Zonation software. Our study area was in Evo, southern Finland, covering a mosaic of managed and conserved forests. We produced the feature layers describing the forest characteristics using high-resolution remote sensing datasets, object-based mapping methods, and forest site type data. We found that increasing the resolution above the 16 m baseline resolution resulted in substantial errors. The conservation errors were the highest for rare features related to European Aspen, whereas the common features related to dominant tree species could benefit from the growth of cell size. We conclude that adequate spatial resolution is a prerequisite for efficient conservation prioritization, and that the size and spatial distribution of the features affect the resolution requirements.

**Supplementary Information:**

The online version contains supplementary material available at 10.1007/s13280-023-01885-6.

## Introduction

Land use planning is highly dependent on spatial data sets. The data provide decision-makers with information on the subject area through key characteristics related to the planning goals. The on-going biodiversity crisis, which strongly connects to the climate crisis, underlines the importance of ecological aspects as part of all land use planning, such as avoiding ecological impacts in development projects and the planning and designation of new protected areas (Montanarella et al. 2018).

Conservation prioritization is an analytical step of systematic conservation planning (Sarkar and Margules 2002). It identifies the priority areas for conservation across the target landscape by finding that set of sites that together maximize conservation outcomes for all included biodiversity features, such as species, habitats, and other relevant indicators. An important part of spatial prioritization is cost-effectiveness, i.e., maximizing conservation benefits with minimal cost or land area (Moilanen et al. 2009; Kukkala and Moilanen 2013). In conservation prioritization, like in all land use planning, the spatial resolution defines the level of detail that can be achieved with spatial data. The requirements for the resolution vary, depending on the purpose of the planning. Hence, the resolution and quality of the data affect the use and interpretation of spatial data sets and eventually the results of the analysis (Araujo et al. 2005; Rondinini et al. 2006). In ecological datasets the effect of spatial resolution varies by the scarcity of the phenomena. The more detailed the input data, the smaller patterns can be distinguished from the landscape. On the other hand, very detailed data can be impossible to gather due to higher data acquisition and processing costs, and the work can be left undone because of this. Spatially explicit ecological data that cover large areas at resolution relevant to land use planning are not, however, easy to obtain. Most biological survey data are based on point observations that cover only a small fraction of subject areas, tend to be spatially biased, and do not provide information from unsurveyed areas (Boakes et al. 2010; Anderson 2012).

Biodiversity (BD) is typically mapped and monitored using indicators, because the phenomenon itself is often too complicated to measure in detail with the available resources (Sakar et al. 2002). The BD indicators are typically phenomena, species, or other structural features that indicate the site’s potential for hosting other species (Ćosoviċ et al. 2020). For example, in boreal forests, the presence of European aspen (*Populus tremula* L.) and the amount of deadwood are key indicators for the level of biodiversity (Harmon et al. 1986; Kuuluvainen 1994; Krankina and Harmon 1995; Kivinen et al. 2020).

RS-based forest inventory systems have been used already for decades in producing landscape-level forest resource data (see, e.g., Tomppo 2006; Kangas et al. 2018). However, the coarse resolution of the landscape-level data dissolves the scattered BD indicators into the forest matrix and the important details are lost. This is problematic for ecological mapping, especially as the ecologically significant remnants of natural forest structure are scarce within the mosaic of managed forests (Korhonen et al. 2021). Like the large-scale methods, also methods used in collecting compartment-level data for operational forest planning are typically based on field samples that are generalized for larger landscapes using various RS data (e.g., Packalén and Maltamo 2007). Such operational data do not adequately represent the scarce ecological features, as the economically significant trees are typically emphasized in the collection of field data (Suomen Metsäkeskus 2021). Hence, the data produced using RS methods are often either too coarse or imprecise for making ecologically sound land-use decisions.

Within the last decade, very high-resolution RS data, such as airborne LiDAR and drone imagery, have introduced new possibilities in mapping forest structure and BD indicators (Polewski et al. 2018). Coupled with powerful machine learning algorithms and various spectral datasets, these mapping methods have shown to be able to produce detailed spatial data at the level of individual trees (Sothe et al. 2020). However, the object-based mapping methods typically suffer from bias caused from not detecting all objects (error of omission) and, on the other hand, falsely detecting objects that do not really exist (error of commission) (see, e.g., Korpela et al. 2007). The question remains whether the enhanced data on forest structure brings enough advantages to compensate for the problems related to detection bias.

The concept of spatial resolution is a well-studied subject in ecology (Poiani et al. 2000; Rahbek 2005). However, many of the studies focus on distribution of individual species rather than the overall ecological value. Hence, there is a limited number of studies focusing on the effects of spatial resolution on conservation decisions. Also, majority of the studies focusing on the overall conservational value date back several decades, which means that the possibilities of acquiring high-resolution data by means of RS have improved significantly. Studying the effect of sampling unit size on the variability of species richness in North America, Stoms (1994) showed that using larger cell sizes lowered the reported species richness indices. Jantke et al. (2013) studied the efficiency of wetland conservation at different spatial scales over Europe and showed the link between the increase of input data resolution and the enhanced conservation efficiency. More recently, Delangre et al. (2018) studied the role of resolution and shape of the mapping units in habitat suitability models and showed that resolution has a significant effect on model performance, exceeding that of the shape of mapping units. Still, the spatial resolution in all reported studies was rather coarse when compared to the level of detail available in current RS datasets.

The aim of this study is to investigate the effect of the spatial resolution in spatial prioritization analysis of a fragmented forest landscape. To achieve this, we use detailed airborne RS data sets and novel machine learning methods with Zonation 5 software to produce a priority ranking of forest to support the planning of a hypothetical conservation plan. Our focus was not on the accuracy of state-of-the-art object-based methods, but on the effect their output detail has on conservation outcomes.

We executed six prioritization analyses, each with the same input features but with different resolutions and compared the results to answer the following questions:

1. How does the resolution affect the spatial and conservation error of prioritization? (R1)
2. Which input features are the most robust to changes in spatial resolution? (R2)
3. What type of forests benefit the most from detailed data? (R3)

## Materials and methods

### Study area

**Fig. 1:**
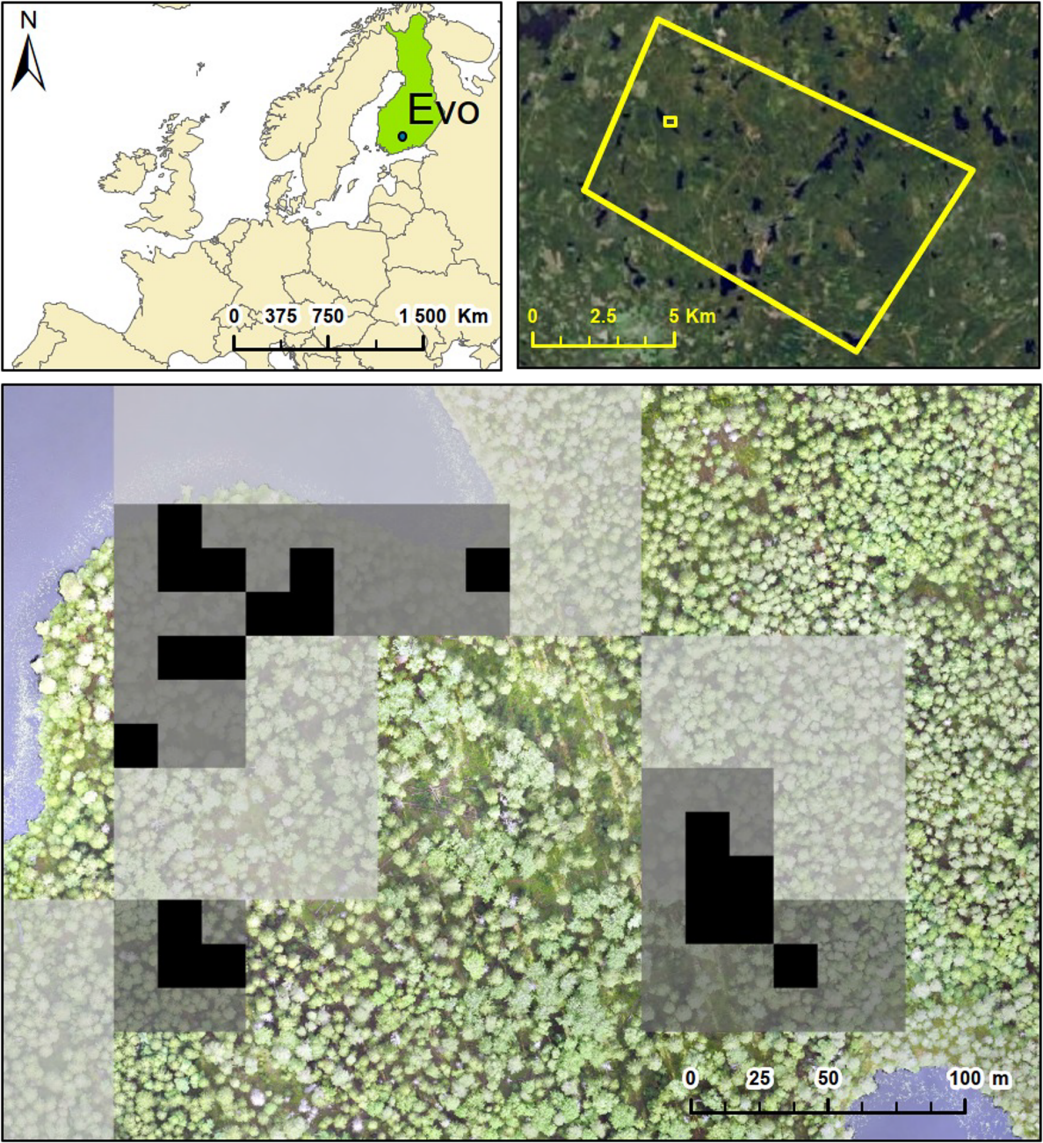
**Upper panels** The Evo study area in southern Finland. **Lower panel** An outtake of aspen occurrence at 16 m (black), 48 m (medium gray), and 96 m (light gray) resolutions (Upper panel left: Made with Natural Earth. Upper panel right: NLS Finland image archives, 12/2022. Lower panel: UEF-drone lab)

Our study area was in southern Finland consisting of a mosaic of intensively managed forest, recreational forests, and forest with strict conservation status (Fig. 1). The study area covers 84 km^2^ and has been measured intensively both in the field and by various remote sensing methods (see, e.g., Mäyrä et al. 2021). The forests in the area are dominated by conifers Norway spruce (*Picea abies* (L.) Karst) and Scots pine (*Pinus sylvestris* L.) representing 40% and 35% of the total volume, respectively. Together, silver birch (*Betula pendula* Roth) and downy birch (*Betula pubescens* Ehrh.) represent 17% and European aspen 5% of the total volume. The study area is rather fertile as over 30% of it consists of herb-rich areas (herb-rich forests and herb-rich heath forests) and more than 50% of mesic heath forests (Table 1). Sub-xeric heath forest and areas with even lower productivity are rather scarce, representing only about 17% of the area. The area has a long history in forestry as the first forestry school in Finland was founded there in 1862. However, in addition to managed forests the study area covers two valuable old forest areas (Kotinen and Sudenpesänkangas) that have had very little human impact.

**Table 1:** Forest site type classes

Used site fertility classification	Class abbreviation	Original fertility classes	Share of the total land area (%)
Herb rich	f1	Herb-rich forests, herb-rich heath forest	32.4
Mesic	f2	Mesic heath forest	51.1
Sub-xeric	f3	Sub-xeric heath forest	15.6
Low productive	f4	Xeric heath forest and barren heath forest. Additionally, the class includes rock outcrop and scree habitat types	0.9

Managed, recreational, and strictly conserved areas cover 24%, 54%, and 21% of the study landscape, respectively. For capturing the site types of the study area, we utilized the compartment-level forest resource data collected by the Finnish Forest Centre (Finnish Forest Centre 2022). The original data divides the forest soils into seven fertility classes, ranging from mesic groves to dry sands and rock. Because of the scarcity of the most nutrient rich and the most barren sites types, we combined the classes on both ends of the spectrum. Thus, we divided the area in four classes describing the site fertility (Table 1).

### Data

We utilized several datasets that were originally collected and processed for other studies or operational forest management. We derived the forest data from object-level measurements of both living and dead trees, which allowed a detailed description of small-scale variation in the forest structure. The high-resolution RS data enabled detailed description of the key indicators of forest biodiversity, such as location, size, and species of trees in the dominant canopy layer, as well as downed deadwood (DDW).

We used existing vector format tree maps produced for Mäyrä et al. (2021) as a basis for our object-based data on standing trees. The location, height, and tree species were determined for all the 2.4 million treetops in the study area using a fusion of airborne laser scanning (ALS) data and airborne hyperspectral data (for details see Mäyrä et al. 2021). The existing tree-level data were determined for the four most frequent tree species groups in the area. Three of the groups consisted of individual tree species (Norway spruce, Scots pine, and European aspen), whereas the fourth group was a combination of the two birch species (silver birch and downy birch). Standing dead trees were not separated from the living trees.

**Fig. 2:**
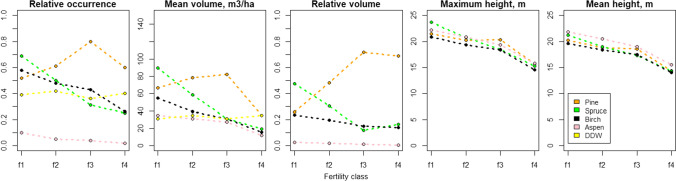
Features occurrences at 16 m cell size. The figure shows the distribution of forest characteristics between the four forest site type classes (f1 = Herb rich, f2 = Mesic, f3 = Sub-xeric, f4 = Low productive)

For the purposes of this study, we processed the data further by adding data on diameter at breast height (DBH) and stem volume. First, we used the ALS-derived tree height to estimate the DBH with existing species-specific equations (Kalliovirta and Tokola 2005). Height and DBH were further used for estimating the stem volumes with species-specific volume equations by Laasasenaho (1982). Figure 2 shows the relative occurrence of species and distributions of other features between the four forest site type classes (listed in Table 1).

As comprehensive DDW data were not available for the study area, we estimated the presence of DDW with dense ALS data and 3D object detection. We used the method introduced in Heinaro et al. (2021) that is based on detecting linear objects from height classified ALS point clouds. The method detects the fallen trunks directly from the point clouds and derives them into estimates of DDW instances with diameter, length, and volume.

### Data processing and analysis

We first transformed all data layers into regular 16 m × 16 m grid cells. The cell values were calculated using the object, i.e., tree-level data within each grid cell. For the living trees, we calculated total volume (Vol), height of the tallest tree (*H*_max_), and mean height of the trees (*H*_mean_) within the grid cells. The attributes related to living trees were calculated separately for the four species groups used in the study. For the DDW, we calculated the total volume of DDW instances within the grid cells. The DDW instances occupying several grid cells were divided correspondingly. After calculating the grid-level attributes, we connected them with the four site type classes (Table 1). Each tree species class formed its own data layer in all four site type classes.

Finally, we aggregated the original data layers for 32 m, 48 m, 64 m, 80 m, and 96 m grid cells. The 16 m cell size is used in Finland for operational forest data (e.g., Mäkisara et al. 2022), whereas the largest size has been used before in nation-wide prioritization tasks (e.g., Mikkonen et al. 2018; Forsius et al. 2021). For the larger cells, we calculated the mean height as an average, maximum height as maximum, and total volume as a sum of the 16 m cells within them. As a result, we had a total of 312 data layers describing the attributes of living trees (288) and DDW (24) describing the spatial forest characteristics of the study area (Table 2, Appendix S1). As all coarser data were generated from the 16 m data, the inevitable uncertainties in the object-based mapping of forest structure should not have affected the results between different resolutions.

**Table 2:** Biodiversity variables and resolutions used to compile input data for the conservation prioritization. Spatial maps of biodiversity features (height and volume of dominant tree species and dead wood volume, *n* = 13) were prepared separately for each site class (*n* = 4) and resolution (*n* = 6), resulting in a total of 312 data layers

Tree size (3)	Hmax, Hmean, and Vol
Tree species (4)	Spruce, Pine, Birches, and Aspen (living trees only)
Volume of downed deadwood (1)	DDW (no species information)
Site classes (4)	f1, f2, f3, and f4
Data resolutions (6)	16 m, 32 m, 48 m, 64 m, 80 m, and 96 m

Next, we identified priority forest areas for a hypothetical conservation plan. The study area was prioritized using Zonation 5 spatial prioritization software (Moilanen et al. 2022). Zonation produces a nested hierarchical priority ranking of spatial units, here grid cells, based on their importance for conservation. Through an iterative optimization process, the software seeks to find a priority ranking of cells that maximizes the representation of all input biodiversity features in the top ranked grid cells. The optimization utilizes core concepts of systematic conservation planning such as complementarity and irreplaceability (Kukkala and Moilanen 2013) to find the most cost-effective solution (or area-effective, if no cost data is available). Consequently, the top ranked areas typically cover core or high-quality habitats of all input features (see, e.g., Kujala et al. 2018). Additionally, Zonation reports information on the fraction of each input feature’s total distribution that is protected by any top ranked fraction of the grid cells. All together six analyses were done, each with the same number of input features (*n* = 52) but at six different resolutions (See Appendix S1). During the prioritization, feature-specific benefits (vj) of protecting a grid cell *i* were treated additive (Moilanen 2007). For each feature *j*, we used a benefit function to describe the value of protection vj as a function of the accumulating fraction rj of feature *j*’s total distribution that is protected. vj (rj) is a power function, where the parameter *z* defines the shape of the function. We set *z* = 0.25 for all biodiversity features, following the well-established species-area relationship.

From each of the six spatial prioritization results, we selected the top ranked 2% and 10% of grid cells to represent our conservation plan. The 10% was based on the EU biodiversity strategy 2030 target of strict protection (European Commission 2020). In addition, we selected the 2% to understand if there are additional effects on the most critically important areas.

For defining the spatial error, we used the prioritization results based on the 16 m resolution data as our baseline. We then compared the top 2% and 10% priority areas identified at the coarser resolutions with those of the baseline. From the comparisons, we first defined the relative area shared with the baseline map, i.e., intersection of the two prioritizations. In addition, we defined the error of omission for all five resolutions. For conservational error, we calculated the relative loss or gain in protection of input features that happens as the selection of priority areas are based on increasingly coarser data. We did this by comparing the total sums of each feature (i.e., Hmax, Hmean, and Vol) that is protected by the different priority solutions, calculated using the baseline (16 m) data.

## Results

### Spatial error

**Fig. 3:**
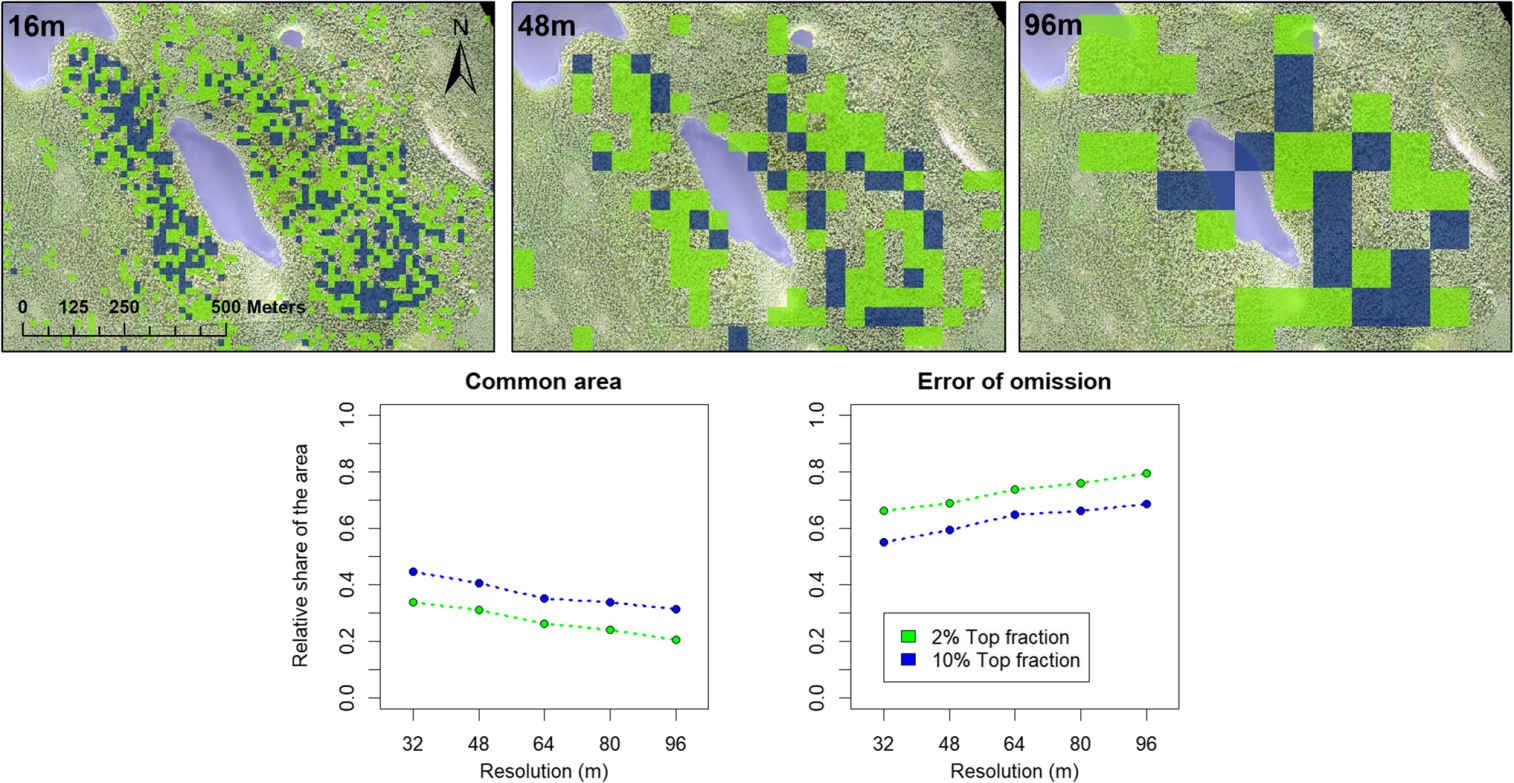
**Upper panel** An outtake from the study area showing the 2% (green) and 10% (dark blue) prioritized top fractions at 16 m, 48 m, and 96 m cell size. The 2% top fraction is included in the 10% top fraction. **Lower panel** Detected spatial error in relation to the decrease of resolution. The relative value of common area and error of omission sum up to 1. Background image: RGB-drone data UEF-drone lab

The overlap with the baseline priority areas decreased with cell size, both for 2% and 10% top fractions (Fig. 3). The overlap of the 2% top fraction varied between 0.34 (32 m × 32 m resolution) and 0.21 (96 m × 96 m), whereas for the 10% top fraction the overlap varied between 0.45 and 0.31. The decrease in the overlap was highest between baseline and the next finest 32 m × 32 m resolution, although the greatest drop in the overlap took place already during this first resolution decrease (Fig. 3). The error of omission increased steadily with increasing cell size.

### Conservation error

**Fig. 4:**
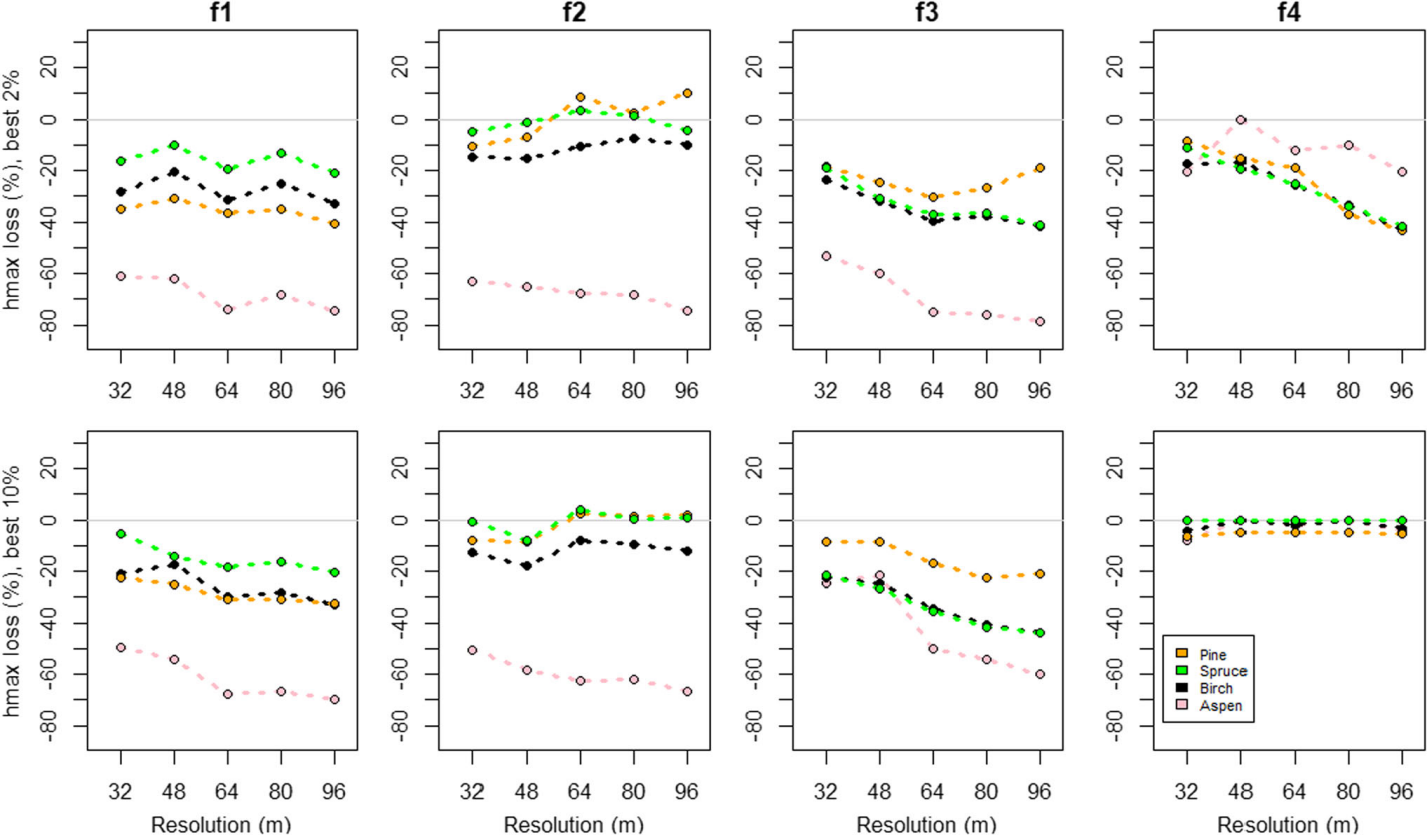
The relative change of height features’ values that are protected by the priority areas under each data resolution. Here, Hmax describes the height of the tallest trees within the map cells. The top row presents the variation in the top 2% fraction and the lower row in the top 10% fraction

**Fig. 5:**
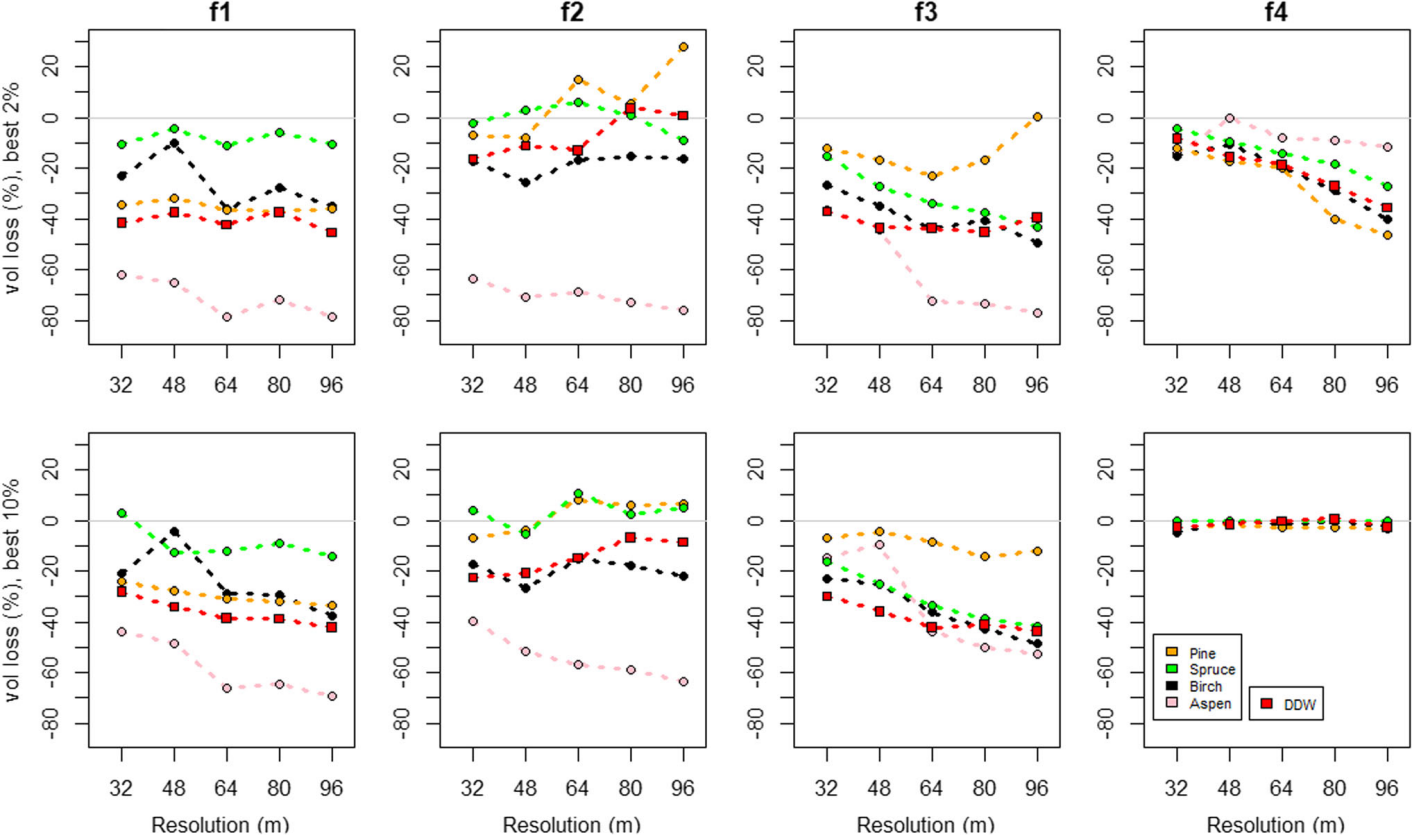
The relative change of volume features’ values that are protected by the priority areas under each data resolution. Here vol describes the volume of each tree species and DDW. The top row presents the variation in the top 2% fraction and the lower row in the top 10% fraction

We report the results for conservation error separately for height features (Fig. 4) and volume features (Fig. 5). For height features, only results for feature *H*_max_ are shown, as the outcomes for *H*_max_ and Hmean behaved similarly with the increase of the cell size. Results for feature *H*_mean_ are given in Appendix S2.

Overall, the decrease of spatial accuracy (i.e., increasing cell size) had a negative impact on the conservation outcome for features (Figs. 4, 5, Appendices S2 and S3). In most cases, the priority areas protected less of their values within the priority areas, than what could have been protected with a higher resolution data. There were a few cases that stood out. For biodiversity features found on forest site type class 4, which was the rarest site type, the 2% top fractions show an increasing conservation error with resolution in both height and volume features, but in the 10% top fraction the error remains marginal and steady at all cell sizes and the differences between feature types are small.

In site type class 2, both the height and volume features of the dominant tree species, i.e., pine and spruce, show a positive trend between the increasing cell size and decreasing conservation error. In the 2% top fraction, the conservation error for height features of pine, spruce, and birch decreases with cell size (Fig. 4). For pine and spruce, the outcomes with coarser data were at times even greater than the 16 m baseline. In the 10% top fraction, the errors for pine, spruce, and birch are generally higher but still close to or even slightly above the baseline. In volume features, the trends are similar but even more positive for pine and spruce (Fig. 5). In all site types the effect of increasing resolution varied between different features. Aspen features suffered the most from the increase of cell size. While these features lost 20%-60% from their highest attainable protection already when moving from baseline to 32 m cell size, the loss increased steadily further, peaking at about 75% (Vol feature at 96 m cell size, 2% top fraction). For the DDW, the effect of cell size was twofold. The relative loss from the baseline is significant, but in forest site type class 2, the increase in cell size resulted in better outcomes than the baseline.

## Discussion

In this study, we explored the effect of spatial resolution on spatial prioritization-based conservation outcomes in a boreal forest site. Our results show that the spatial error increases drastically when resolution is lowered (R1). The relative share of common area between the baseline and coarser resolution solutions dropped to 0.35–0.45 (2% and 10% top fractions, respectively) already at the first cell size increase from 16 × 16 m to 32 × 32 m (Fig. 3). The share of common area continued to decrease with increasing cell sizes, but slower. We believe that the relative increase in the cell area plays an important role here. Increasing the cell size from 16 × 16 m to 32 × 32 m results in four times larger cell extent, whereas further increases in cell size have much lower relative effect on the cell extent. Also Arponen et al. (2012) reported low overlap between the top priority outcomes in prioritization analyses conducted at different resolutions.

The increase in spatial error also led to higher conservation errors, although there was a lot of variation between the features (R1). Typically, the spatially more common features were less affected by the increasing cell size and even benefited from it, whereas more scarce features were clearly lost. The effect of scarcity can be seen especially for aspen. Having the lowest relative occurrence and relative volume and being a species that occurs only sporadically, often as single trees, in the study area, information about aspen-specific biodiversity features became lost when cells were aggregated into larger units. Unlike dead wood and features of other tree species, aspen-specific features had a declining trend in all forest site type classes and both top fractions, the only exception being the Low productive site type class (f4) in the 10% top fraction.

In our study system, both the tree species and the site type class defined how common or rare a feature was. For example, spruce was one of the most common dominant species in site type classes 1 and 2 (Fig. 2), and in both classes, the conservational error of spruce-specific features remained relatively low (Figs. 4 and 5, Appendix S2). In contrast, spruce was less commonly dominant in site type class 3, and in this class the increasing cell size increased the conservation error of spruce-specific features. On the other hand, forest site type class itself can be rare. In our study, the site type class 4 covered only 0.9% of the total study area. Since the prioritization aimed to capture core occurrences of all features within the priority areas, the few locations of site type 4 became selected at rather high frequency (Appendix S4). This led to all species behaving rather similarly within site class 4, especially within the 10% top fractions, where nearly all of the cells of this site class were selected in the priority areas (Figs. 4 and 5. Appendix S2). According to our results, the forest areas hosting scattered and small-scale features tend to be substituted by areas hosting more common features in conservation plans based on coarser data (R2 and R3). We believe this to be driven by two processes. First, the spatial aggregation of feature’s occurrences defines how well information about its presence is carried through to higher cell sizes. This is highlighted by the differences between features related to aspen and site type class 4, which show that rare aspen features with sporadic occurrence, tend to be lost in the cell aggregation and therefore suffer from much higher conservation error in comparison to the rare site types forming continuous areas, which are simpler to aggregate into larger cell sizes. Second, with coarser resolutions the number of selectable cells is lower within the landscape. Although the remaining rare features still drive the selection of cells as priorities, at coarser resolution it becomes impossible to select the rare and scattered features without bringing in large amounts of the common features (Fig. 1). From this follows, that the most common features can even benefit from the increasing cell size as the prioritization process actually selects more of these features from the landscape compared to the baseline. For example, the amount of protected pine features at site type class 2 rose well above the baseline (Figs. 4 and 5, Appendix S2).

In this study, the scarce forest features were represented by aspen features (*H*_max_, *H*_mean_, and Vol) and DDW features. In the analyses, the aspen features behaved as expected whereas the behavior of the DDW features was somewhat unexpected. The DDW features’ response to the increasing cell size resembles that of the common tree species (Fig. 5), although it is a scarce and scattered phenomenon in the landscape. Prior to the analysis, the correctness of the DDW feature layer was checked by comparing its average volume to the DDW volumes measured from the area in field surveys. Although the mean volumes were rather similar, it seems that the RS-based detection overestimated the occurrence of DDW by making its spatial distribution more even, thus making it a more common feature in the landscape. The ALS-based DDW mapping method has been reported to work best with large diameter trunks (Heinaro et al. 2021) that contribute the most for the total volume. Although the falsely detected DDW instances, i.e., errors of commission are typically small in size, they build up in larger cell sizes and affect the total volume. As the method seems to work best on large diameter trunks, it would be interesting to use it for mapping only the occurrence of the largest DDW diameter classes. In general, this study did not consider the uncertainties related to remote-sensed forest attributes. However, if the results were to be used for, e.g., planning of new conservation areas, all RS-based forest attribute maps should be validated using adequate reference data.

Scarce features suffered the most from the increase of cell size. Hence, we argue that any rare phenomenon is likely to disappear into the forest matrix with large cell sizes. Lehtomäki et al. (2015) proposes that the data collected primarily for operational forest planning would be informative in the context of spatial conservation prioritization. Unlike the operational forest data that utilizes statistical models to generalize field-measured attributes with RS data (see, e.g., Brosofske et al. 2014), the input features in this study based on direct detection and identification of forest features from RS data. According to our results, even detailed object-level data (i.e., tree and deadwood maps) will not guarantee that scarce and scattered phenomena will be present in the final prioritization results, if the spatial resolution of the final input feature is too coarse for the phenomenon. Such phenomena require a finer resolution than rare but strongly clustered phenomena. Hence, the spatial distribution of the phenomenon affects its chance to be included in conservation schemes.

Ecological features such as local species diversity may result in biased estimates on very fine resolutions (Noss 1990). Hence, there are trade-offs between optimal resolutions for mapping different features. Nevertheless, the cost-efficiency of defining the ecologically most significant areas still suffers from coarse resolution. Coarse resolution data introduces spatial uncertainty to conservation plans, as part of the scarce resources may be misplaced to protect well fairing biodiversity features while rare ones are missed.

As Zonation software only considers the location and relative abundance of input features (Kujala et al. 2018), the results of this study are applicable to all forest ecosystems hosting rare and scattered features that are important for biodiversity. However, the definition of high or coarse resolution varies between ecosystems as it depends on the extent of the target features. The resolution has a significant effect on the qualities of the prioritized forest areas and on the conservation outcome for the target features.

As spatial datasets from rare and scattered phenomena are challenging to produce for high resolutions, the future research should focus on how to produce high-resolution spatial data sets efficiently. Object-based techniques and state-of-the-art remote sensing material already make this possible for the dominant trees (Kaartinen et al. 2012; Dalponte and Coomes 2016). In contrast to the area-based estimates relying on statistically sound plot-level data, object-based methods do not produce unbiased estimates, as all objects are rarely spotted from the RS data. However, only the object-based methods can capture the fine scale variation of the scarce features that are needed in effective spatial conservation prioritization.

## Conclusions

We conclude that prioritizations made with coarse resolution are biased towards the dominant forest types and features in the landscape. Using large cell sizes favors the features that are common and evenly distributed in the landscape. To capture the rare, scattered, and clustered features from the landscape, input features used in prioritizations should be based on spatial data capable of capturing the smallest mappable features significant for the prioritization. High resolution of input features enhances the inclusion of rare and scattered forest features and thus the overall quality of spatial prioritizations in fragmented forest landscapes. High-resolution RS data and object-based methods offer a means for mapping ecological key components in detail for the needs of conservation prioritization.

Still, the twofold nature of resolution remains. As this research has shown, high-resolution input features help maintaining small-scale key components of biodiversity. On the other hand, overly high resolution also results in fragmented output that, e.g., lowers the resilience of conservation areas. Hence, the overall goal of the analysis should always set the final resolution of input features. However, it is important to keep in mind how the chosen resolution affects ecological features with different spatial distribution.

### Supplementary Information

Below is the link to the electronic supplementary material.

Supplementary file1 (PDF 867 KB)
