# Supplementary material for: Input data resolution affects the conservation prioritization outcome of spatially sparse biodiversity features
Source: Ambio. 2023 Jun 2;52(11):1793–803. doi: 10.1007/s13280-023-01885-6 (PMC10562354; doi:10.1007/s13280-023-01885-6)
Supplement: Supplementary file 1 — Supplementary file1 (PDF 867 KB) [file 13280_2023_1885_MOESM1_ESM.pdf]

Ambio

Electronic Supplementary Material

Title: **Input data resolution affects the conservation prioritization outcome of spatially sparse biodiversity features**

Authors: Topi Tanhuanpää, Ninni Mikkonen, Heini Kujala, Einari Heinaro, Janne Mäyrä, Timo Kumpula

Appendix S1. The number of cells in each Zonation input feature at 16m - 96m cell sizes.

Forest site type class	Pine (Hmax, Hmean, Vol)	Spruce (Hmax, Hmean, Vol)	Birch (Hmax, Hmean, Vol)	Aspen (Hmax, Hmean, Vol)	DDW (Vol)	Resolution
F1	52063	69084	58177	9822	38404	16m
F2	95824	78755	75847	8343	65075	
F3	38214	15084	20440	1924	17094	
F4	1676	716	717	43	1113	
F1	21697	24255	24098	6776	19603	32m
F2	34284	31232	32945	6128	31614	
F3	12328	7421	9593	1433	8719	
F4	705	379	429	37	606	
F1	12598	13224	13508	5109	11986	48m
F2	18736	17786	18753	4985	18525	
F3	6465	4679	5707	1170	5394	
F4	436	262	294	35	395	
F1	8426	8585	8806	3988	8090	64m
F2	12163	11798	12325	4103	12229	
F3	4159	3350	3861	1011	3719	
F4	315	199	225	32	286	
F1	6092	6176	6315	3249	5875	80m
F2	8610	8470	8757	3448	8647	
F3	2980	2518	2827	852	2730	
F4	249	162	187	32	226	
F1	4663	4695	4803	2684	4544	96m
F2	6487	6450	6571	3022	6504	
F3	2223	1987	2162	760	2119	
F4	205	139	160	26	193	

Appendix S2. The relative change in the amount of *Hmean* features' values that are protected by the priority areas under each data resolution. Here *Hmean* describes the mean height of the trees within the map cells. The top row presents the variation in the top 2% fraction and the lower row in the top 10% fraction.

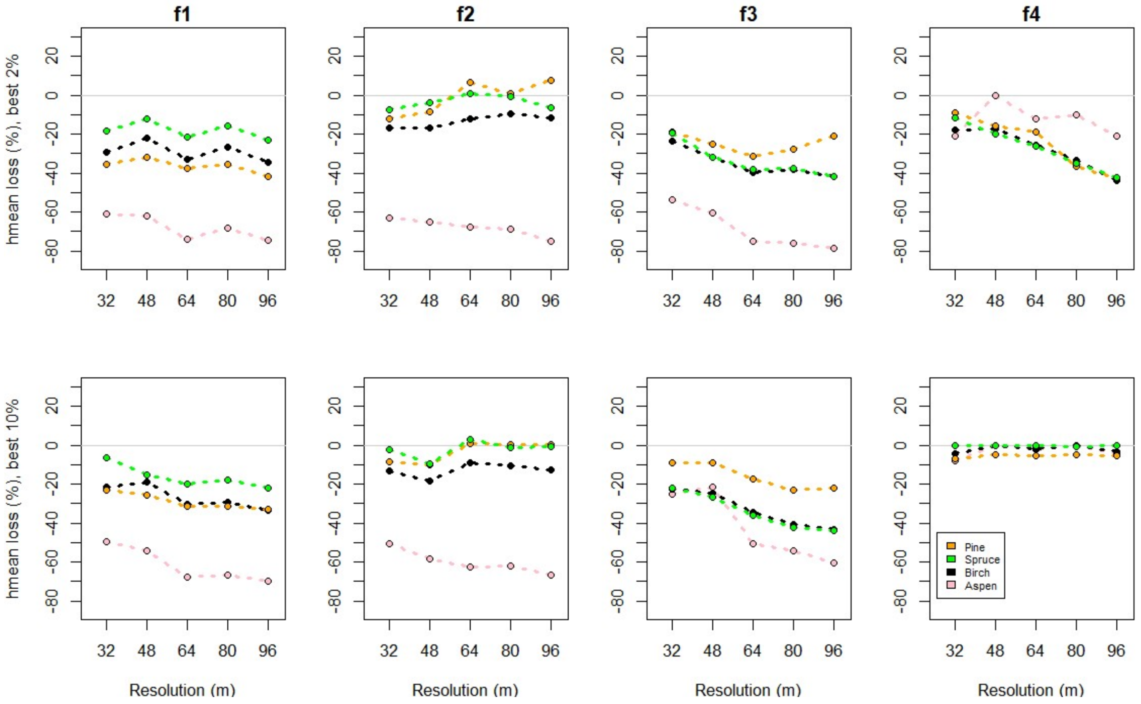

Appendix S3. Changes (%) in feature sums protected by the priority areas between each data resolution and the 16m baseline. In feature names asp stands for aspen, bir for birch, pin for pine, spr for spruce, and ddw for downed deadwood. f1-f4 stand for forest site type classes 1-4, respectively. Hmax stands for the maximum tree height, hmean the mean tree height, and vol the volume related to the feature.

**Cell size**	**32 m**		**48 m**		**64 m**		**80 m**		**96 m**	
**Fraction**	*2 %*	***10 %***	*2 %*	***10 %***	*2 %*	***10 %***	*2 %*	***10 %***	*2 %*	***10 %***
asp_f1_hmax	-60.9	**-49.7**	-61.9	**-54.2**	-74.0	**-67.7**	-68.4	**-66.7**	-74.6	**-70.0**
asp_f1_hmean	-60.9	**-49.8**	-62.0	**-54.3**	-74.0	**-67.7**	-68.4	**-66.7**	-74.5	**-70.0**
asp_f1_vol	-62.2	**-44.0**	-65.3	**-48.7**	-78.7	**-66.1**	-71.7	**-64.4**	-78.7	**-69.3**
asp_f2_hmax	-63.0	**-50.7**	-65.0	**-58.4**	-67.5	**-62.6**	-68.5	**-62.0**	-74.8	**-66.7**
asp_f2_hmean	-63.1	**-50.8**	-65.2	**-58.6**	-67.6	**-62.7**	-68.6	**-62.1**	-74.8	**-66.7**
asp_f2_vol	-63.6	**-39.4**	-71.1	**-51.5**	-68.6	**-56.7**	-73.0	**-59.1**	-75.9	**-63.4**
asp_f3_hmax	-53.3	**-24.8**	-59.9	**-21.3**	-75.1	**-50.3**	-76.2	**-54.2**	-78.6	**-60.2**
asp_f3_hmean	-53.7	**-25.1**	-60.2	**-21.6**	-75.2	**-50.4**	-76.3	**-54.3**	-78.7	**-60.3**
asp_f3_vol	-36.8	**-14.5**	-44.2	**-9.4**	-72.4	**-44.1**	-73.7	**-50.0**	-76.9	**-52.7**
asp_f4_hmax	-20.5	**-7.9**	0.0	**0.0**	-12.1	**-2.1**	-10.0	**0.0**	-20.6	**-4.1**
asp_f4_hmean	-20.9	**-8.0**	0.0	**0.0**	-12.2	**-2.1**	-10.1	**0.0**	-20.9	**-4.2**
asp_f4_vol	-14.3	**-4.9**	0.0	**0.0**	-7.8	**-1.3**	-9.2	**0.0**	-11.7	**-2.7**
bir_f1_hmax	-28.2	**-21.1**	-20.4	**-17.3**	-31.5	**-29.7**	-25.3	**-28.3**	-32.8	**-32.8**
bir_f1_hmean	-29.4	**-21.6**	-22.1	**-18.6**	-32.8	**-30.6**	-26.7	**-29.2**	-34.3	**-33.5**
bir_f1_vol	-23.1	**-20.8**	-9.9	**-4.5**	-36.1	**-28.7**	-27.6	**-29.5**	-35.1	**-37.6**
bir_f2_hmax	-14.9	**-12.9**	-15.5	**-17.6**	-10.7	**-8.2**	-7.6	**-9.8**	-9.8	**-12.0**
bir_f2_hmean	-16.7	**-13.4**	-17.0	**-18.3**	-12.2	**-9.0**	-9.3	**-10.7**	-11.7	**-12.7**
bir_f2_vol	-17.5	**-17.2**	-25.5	**-26.9**	-16.7	**-15.0**	-15.2	**-18.1**	-16.4	**-22.0**
bir_f3_hmax	-23.8	**-22.4**	-31.7	**-24.4**	-39.7	**-34.7**	-37.9	**-41.0**	-41.7	**-43.6**
bir_f3_hmean	-23.8	**-22.5**	-31.6	**-24.6**	-39.6	**-34.7**	-37.9	**-41.0**	-41.6	**-43.5**
bir_f3_vol	-26.5	**-23.1**	-34.8	**-25.4**	-44.0	**-36.1**	-40.8	**-42.7**	-49.8	**-48.4**
bir_f4_hmax	-17.6	**-4.5**	-17.0	**0.0**	-25.6	**-1.6**	-33.4	**-0.4**	-43.4	**-3.1**
bir_f4_hmean	-17.6	**-4.5**	-17.2	**0.0**	-25.8	**-1.6**	-33.6	**-0.4**	-43.7	**-3.1**
bir_f4_vol	-15.1	**-4.7**	-10.7	**0.0**	-19.5	**-1.6**	-28.8	**-0.1**	-40.0	**-3.0**
ddw_f1_vol	-41.6	**-28.0**	-37.5	**-34.0**	-42.2	**-38.4**	-37.3	**-38.6**	-45.4	**-42.1**
ddw_f2_vol	-16.3	**-22.5**	-11.0	**-20.8**	-12.8	**-14.8**	3.8	**-6.7**	0.8	**-8.5**
ddw_f3_vol	-37.2	**-29.7**	-43.6	**-35.8**	-43.7	**-42.1**	-45.3	**-41.0**	-39.4	**-43.6**
ddw_f4_vol	-8.2	**-2.6**	-15.4	**-1.3**	-18.6	**-0.1**	-27.0	**0.7**	-35.5	**-2.4**
pin_f1_hmax	-34.8	**-22.5**	-30.8	**-25.1**	-36.5	**-30.9**	-34.8	**-30.6**	-40.8	**-32.2**
pin_f1_hmean	-35.6	**-22.9**	-31.7	**-25.6**	-37.4	**-31.6**	-35.6	**-31.4**	-41.9	**-32.9**
pin_f1_vol	-34.3	**-24.2**	-31.8	**-27.6**	-36.3	**-31.0**	-37.3	**-31.9**	-36.1	**-33.3**
pin_f2_hmax	-10.7	**-7.8**	-6.9	**-8.7**	8.7	**2.3**	2.4	**1.3**	10.4	**1.9**
pin_f2_hmean	-12.3	**-8.5**	-8.4	**-9.8**	6.5	**1.1**	0.6	**0.1**	7.6	**0.5**
pin_f2_vol	-7.0	**-6.9**	-8.2	**-3.6**	15.1	**8.3**	5.4	**6.2**	27.7	**6.3**
pin_f3_hmax	-18.4	**-8.5**	-24.6	**-8.6**	-30.4	**-16.6**	-26.8	**-22.4**	-18.7	**-20.8**
pin_f3_hmean	-19.0	**-8.8**	-25.2	**-9.1**	-31.2	**-17.1**	-28.0	**-22.9**	-20.9	**-21.8**
pin_f3_vol	-12.3	**-6.9**	-16.8	**-4.1**	-23.0	**-8.6**	-16.8	**-14.2**	0.2	**-12.3**
pin_f4_hmax	-8.6	**-6.5**	-15.5	**-4.6**	-18.9	**-5.1**	-36.9	**-4.9**	-43.2	**-5.2**
pin_f4_hmean	-9.0	**-6.8**	-15.7	**-4.8**	-19.0	**-5.2**	-36.7	**-5.1**	-42.9	**-5.3**
pin_f4_vol	-12.1	**-3.0**	-17.1	**-1.7**	-19.8	**-3.0**	-40.5	**-3.0**	-46.5	**-3.5**
spr_f1_hmax	-16.4	**-5.2**	-10.1	**-14.1**	-19.4	**-18.4**	-13.3	**-16.2**	-20.9	**-20.3**
spr_f1_hmean	-18.2	**-6.6**	-12.2	**-15.2**	-21.6	**-19.8**	-15.6	**-17.7**	-23.2	**-21.8**
spr_f1_vol	-10.7	**2.7**	-4.3	**-12.6**	-11.2	**-12.1**	-5.9	**-8.8**	-10.6	**-14.4**
spr_f2_hmax	-5.0	**-0.8**	-1.2	**-7.9**	3.4	**4.2**	1.6	**0.3**	-4.5	**0.8**
spr_f2_hmean	-7.5	**-2.0**	-4.0	**-9.4**	0.7	**2.7**	-0.6	**-1.1**	-6.5	**-0.7**
spr_f2_vol	-2.2	**3.7**	2.9	**-5.5**	6.2	**10.6**	0.9	**2.2**	-9.0	**4.9**
spr_f3_hmax	-18.8	**-21.5**	-30.9	**-26.7**	-37.3	**-35.5**	-36.8	**-41.9**	-41.4	**-44.0**
spr_f3_hmean	-19.7	**-21.9**	-31.9	**-26.9**	-38.1	**-35.8**	-37.6	**-42.2**	-42.0	**-44.1**
spr_f3_vol	-15.2	**-16.5**	-27.4	**-25.4**	-33.8	**-33.7**	-37.9	**-38.8**	-43.5	**-41.6**
spr_f4_hmax	-10.9	**0.0**	-19.3	**0.0**	-25.3	**0.0**	-34.2	**-0.4**	-41.8	**-0.2**
spr_f4_hmean	-11.5	**0.0**	-20.1	**0.0**	-26.1	**0.0**	-35.2	**-0.4**	-42.5	**-0.2**
spr_f4_vol	-4.3	**0.0**	-9.5	**0.0**	-14.1	**0.0**	-18.4	**-0.1**	-27.1	**-0.1**

Appendix S4. Shares of total feature sums protected by the priority areas under each data resolution.

Cell size	16 m		32 m		48 m		64 m		80 m		96 m	
Fraction	*2 %*	*10 %*	*2 %*	*10 %*	*2 %*	*10 %*	*2 %*	*10 %*	*2 %*	*10 %*	*2 %*	*10 %*
asp_f1_hmax	0.6594	0.1645	0.3555	0.0702	0.253	0.0558	0.197	0.0374	0.1962	0.0384	0.1727	0.0327
asp_f1_hmean	0.6585	0.1639	0.353	0.07	0.2526	0.0558	0.1981	0.0376	0.1965	0.0383	0.1728	0.0329
asp_f1_vol	0.8074	0.2667	0.6018	0.1772	0.5005	0.1492	0.3875	0.1173	0.402	0.1309	0.3731	0.0904
asp_f2_hmax	0.6805	0.1938	0.3493	0.0854	0.2552	0.0662	0.2156	0.0431	0.2181	0.0423	0.1936	0.0369
asp_f2_hmean	0.6789	0.1931	0.3466	0.0849	0.2536	0.066	0.2152	0.0427	0.2159	0.0418	0.192	0.0365
asp_f2_vol	0.8456	0.3601	0.66	0.2447	0.5476	0.2034	0.4949	0.1594	0.5248	0.177	0.5033	0.1457
asp_f3_hmax	0.9982	0.6376	0.8937	0.3028	0.7155	0.1906	0.4926	0.1233	0.4432	0.1123	0.3858	0.1046
asp_f3_hmean	0.9981	0.6352	0.8906	0.2985	0.7068	0.1877	0.4892	0.1233	0.4395	0.1092	0.3809	0.1006
asp_f3_vol	0.9996	0.8241	0.9782	0.6286	0.9229	0.5039	1	0.8889	1	1	1	0.9375
asp_f4_hmax	1	1	1	1	1	1	1	1	1	1	1	0.9718
asp_f4_hmean	1	1	1	1	1	1	1	1	1	1	1	0.9702
asp_f4_vol	1	1	1	1	1	1	1	0.9647	1	1	1	0.9968
bir_f1_hmax	0.158	0.0279	0.154	0.0284	0.1523	0.0294	0.138	0.0273	0.1359	0.027	0.1283	0.0255
bir_f1_hmean	0.1612	0.0287	0.1579	0.0294	0.1562	0.0306	0.1427	0.0285	0.1409	0.0283	0.1328	0.0265
bir_f1_vol	0.1683	0.0292	0.1834	0.0376	0.2101	0.0366	0.1624	0.0307	0.1519	0.0302	0.1472	0.0291
bir_f2_hmax	0.1216	0.0218	0.1231	0.0253	0.1262	0.0259	0.1318	0.0251	0.1306	0.0255	0.1236	0.0234
bir_f2_hmean	0.1229	0.0223	0.1256	0.026	0.1299	0.0272	0.136	0.0262	0.1344	0.0265	0.1283	0.024
bir_f2_vol	0.1651	0.0297	0.1925	0.043	0.2101	0.0366	0.1987	0.0374	0.1879	0.0384	0.1769	0.0357
bir_f3_hmax	0.3998	0.0861	0.3019	0.0668	0.2818	0.0577	0.2565	0.0525	0.2441	0.0519	0.2336	0.0504
bir_f3_hmean	0.397	0.0854	0.2981	0.0662	0.2795	0.0581	0.2564	0.0526	0.2458	0.0525	0.2342	0.0507
bir_f3_vol	0.5343	0.1333	0.4749	0.1268	0.4544	0.1133	0.4131	0.1015	0.3722	0.0964	0.35	0.0861
bir_f4_hmax	1	0.9523	1	0.8251	1	0.7304	0.9962	0.6395	0.9924	0.554	0.9909	0.491
bir_f4_hmean	1	0.9506	1	0.8201	1	0.7221	0.9958	0.6299	0.9917	0.5435	0.9897	0.4797
bir_f4_vol	1	0.9827	1	0.9297	1	0.8922	0.9988	0.8268	0.9991	0.7494	0.9975	0.6523
cwd_f1_vol	0.1716	0.0331	0.1643	0.0333	0.1539	0.0321	0.1381	0.0284	0.1324	0.0298	0.1309	0.0329
cwd_f2_vol	0.0883	0.0151	0.098	0.0209	0.1162	0.0255	0.1132	0.0217	0.1267	0.0256	0.1145	0.024
cwd_f3_vol	0.2993	0.061	0.2482	0.0553	0.2473	0.0549	0.2375	0.0515	0.2422	0.0556	0.2144	0.0542
cwd_f4_vol	0.9507	0.687	0.9235	0.6178	0.9311	0.603	0.9168	0.5289	0.936	0.5478	0.8835	0.4304
pin_f1_hmax	0.152	0.0284	0.1569	0.0277	0.1485	0.0289	0.138	0.0273	0.1372	0.0277	0.1311	0.026
pin_f1_hmean	0.1551	0.0292	0.1603	0.0286	0.1518	0.03	0.1414	0.0286	0.1407	0.0289	0.1344	0.0271
pin_f1_vol	0.141	0.0236	0.1488	0.026	0.1402	0.0251	0.1339	0.0238	0.1255	0.0226	0.1241	0.0214
pin_f2_hmax	0.0828	0.0143	0.1099	0.0222	0.1213	0.0246	0.1284	0.0249	0.1273	0.0239	0.1241	0.0236
pin_f2_hmean	0.0852	0.0149	0.1135	0.0232	0.1255	0.0258	0.1325	0.0258	0.1313	0.0248	0.1278	0.0242
pin_f2_vol	0.063	0.0099	0.0894	0.0162	0.1002	0.0171	0.1033	0.0176	0.0994	0.0164	0.1003	0.0188
pin_f3_hmax	0.2129	0.0428	0.2316	0.0476	0.2407	0.0482	0.2311	0.047	0.2254	0.0469	0.2163	0.0469
pin_f3_hmean	0.217	0.0441	0.2371	0.0496	0.2472	0.0503	0.2368	0.0488	0.2318	0.0486	0.2212	0.0484
pin_f3_vol	0.1861	0.0323	0.2041	0.0382	0.2174	0.0382	0.22	0.0351	0.2109	0.0387	0.2112	0.0406
pin_f4_hmax	1	0.619	0.9613	0.5773	0.9313	0.5249	0.9278	0.4812	0.9049	0.4213	0.8899	0.3761
pin_f4_hmean	1	0.6166	0.9576	0.5691	0.9291	0.5197	0.9274	0.4685	0.8936	0.4155	0.8806	0.3666
pin_f4_vol	1	0.6956	0.9959	0.6873	0.9871	0.6262	0.9789	0.6003	0.9736	0.5047	0.9568	0.489
spr_f1_hmax	0.1449	0.0242	0.1533	0.0278	0.1584	0.0309	0.1462	0.028	0.1436	0.0282	0.1327	0.0247
spr_f1_hmean	0.1475	0.025	0.1532	0.028	0.1585	0.031	0.1457	0.0279	0.1431	0.0282	0.1321	0.0247
spr_f1_vol	0.1422	0.0225	0.1724	0.0302	0.169	0.0327	0.1719	0.0314	0.1659	0.0301	0.1523	0.0273
spr_f2_hmax	0.1219	0.0222	0.1348	0.0274	0.1431	0.0302	0.1405	0.0273	0.141	0.0273	0.1297	0.0251
spr_f2_hmean	0.1229	0.0227	0.1332	0.027	0.1411	0.0299	0.1387	0.0269	0.1394	0.0271	0.1281	0.0249
spr_f2_vol	0.1447	0.0267	0.1899	0.0401	0.2044	0.047	0.2156	0.0413	0.2063	0.0383	0.1921	0.0343
spr_f3_hmax	0.4925	0.1101	0.3654	0.0863	0.3383	0.0738	0.2926	0.0638	0.2779	0.0617	0.2514	0.055
spr_f3_hmean	0.4883	0.1099	0.3597	0.0849	0.333	0.0724	0.2875	0.0627	0.2731	0.0606	0.2486	0.0546
spr_f3_vol	0.6554	0.1753	0.5855	0.1719	0.5477	0.1553	0.4952	0.1404	0.4849	0.1357	0.4352	0.121
spr_f4_hmax	1	0.9765	1	0.8392	1	0.7584	1	0.6497	0.9911	0.614	0.9894	0.4928
spr_f4_hmean	1	0.9752	1	0.8321	1	0.7499	1	0.6404	0.9906	0.6042	0.9888	0.4879
spr_f4_vol	1	0.9931	1	0.9481	1	0.927	1	0.835	1	0.8699	0.9994	0.7226
